# Somatic embryogenesis and plant regeneration from transverse thin cell layers of adult peach palm (*Bactris gasipaes*) lateral offshoots

**DOI:** 10.3389/fpls.2022.995307

**Published:** 2022-09-28

**Authors:** Stefanny Campos-Boza, María Vinas, Paul Solórzano-Cascante, Andrea Holst, Douglas A. Steinmacher, Miguel P. Guerra, Víctor M. Jiménez

**Affiliations:** ^1^Centro para Investigaciones en Granos y Semillas (CIGRAS), Universidad de Costa Rica, San Pedro, Costa Rica; ^2^Vivetech Agrociências, Marechal Cândido Rondon, Brazil; ^3^Plant Developmental Physiology and Genetics Laboratory, Department of Plant Science, Federal University of Santa Catarina, Florianópolis, Brazil; ^4^Graduate Program in Agricultural and Natural Ecosystems, Federal University of Santa Catarina, Curitibanos, Brazil; ^5^Instituto de Investigaciones Agrícolas (IIA) and Cátedra Humboldt, Universidad de Costa Rica, San Pedro, Costa Rica

**Keywords:** peach palm, pejibaye, picloram, somatic embryogenesis, thin cell layer

## Abstract

In this work, we report a successful protocol to obtain *in vitro* peach palm (*Bactris gasipaes* Kunth) “Diamantes 10” plants through somatic embryogenesis from transverse thin cell layer (TCL) explants, dissected from three sections (basal, medial, and apical) of lateral offshoots of adult plants cultured on different concentrations of 4-amino-3,5,6-trichloropicolonic acid (picloram). After swelling and development of primary callus in all treatments, without any strong effect of explant origin or picloram concentration, it was possible to observe the formation of embryogenic structures and the exact point from where they developed. Browning was also observed and correlated to the induction treatments, although it was not an impairment for the production of embryogenic structures. Subsequent maturation and conversion of somatic embryos into plantlets allowed their acclimatization 17 months after culture initiation (ACI), which was quicker than previous reports with juvenile tissues (from embryos or seed-germinated plantlets). To the best of our knowledge, this is the first report on peach palm regeneration through somatic embryogenesis from TCL explants from adult plants and could constitute, after fine-tuning the acclimatization stage, a tool for mass clonal propagation of elite genotypes of this open-pollinated crop, as well as for the establishment of conservation strategies of *in situ* gene bank plant accessions endangered due to aging and other threats.

## Introduction

Peach palm, also known as pejibaye, chontaduro, or pupunha (*Bactris gasipaes* Kunth—Arecaceae), is considered the most highly domesticated palm species in the Neotropics and of economic importance in several countries of the American continent (Clement, 1988; Mora-Urpí et al., 1997; Galluzzi et al., 2015), with fruit and palm heart being its most important products found in the market (Clement and Mora-Urpí, 1987; Clement, 2008). The consumption of peach palm fruit was already important during pre-Columbian times (Clement et al., 2004), and its nutritional and functional value has been the subject of recent analyses (dos Santos et al., 2020; da Costa et al., 2022). However, the significant variability found in the composition of fruits from different accessions highlights the importance of having efficient systems for the propagation of selected materials (Hempel et al., 2014; Chisté et al., 2021).

Seed propagation is the usual method for establishing new peach palm plantations. However, due to allogamy and self-incompatibility, the variability usually found in natural and cultivated populations is very high (Clement and Arkcoll, 1984). Performing controlled pollination is challenging due to the tall height that plants reach in the field, which makes it difficult to conduct targeted crosses between genotypes (Mora-Urpí, 1980). Therefore, vegetative propagation has been considered for establishing commercial plantations with selected genotypes. However, the use of offshoots (tillers) has given only partially positive results and at relatively low rates (Flores et al., 2012), while micropropagation has been considered a possibility worth further exploration (Stefenon et al., 2020).

Somatic embryogenesis has been described as the most efficient pathway to obtaining plants in this species. While different explants have been tested, e.g., shoot tips, adventitious buds, leaf primordia, zygotic embryos, inflorescences, and transverse thin cell layers (TCLs) (explants consisting of a few cell layers, usually 0.5–1.0 mm thick, TCLs) (reviewed by Stefenon et al., 2020), it is with the latter that most success and highest reproducibility have been obtained. Effective induction of somatic embryogenesis by using TCL has been reported in many species. Thin explants have shown higher response rates to *in vitro* conditions as compared to more voluminous ones, probably due to a larger surface area in contact with the surrounding environment and, thus, better perceiving chemical and physical stimuli (Teixeira-Da Silva and Dobránszki, 2019).

However, in the peach palm, explants for *in vitro* propagation have been obtained mostly from zygotic embryos or seedlings from seeds germinated *ex vitro* or *in vitro*, which does not meet the objective of clonally multiplying evaluated and selected materials. Only two recent papers mention clonal propagation of peach palms from adult tissues. de Almeida and de Almeida (2006) reported plant regeneration through somatic embryogenesis from isolated adult leaf primordia but without providing quantitative data, while Steinmacher et al. (2007b) used immature inflorescences from adult plants as starting material to obtain only a few plants. Therefore, this work aimed to establish an efficient protocol based on somatic embryogenesis for *in vitro* propagation of peach palm through TCL from offshoot tips of adult plants looking at the mass clonal propagation of elite genotypes and rescue of endangered individuals in *in situ* collections.

## Materials and methods

### Plant material

**FIGURE 1:**
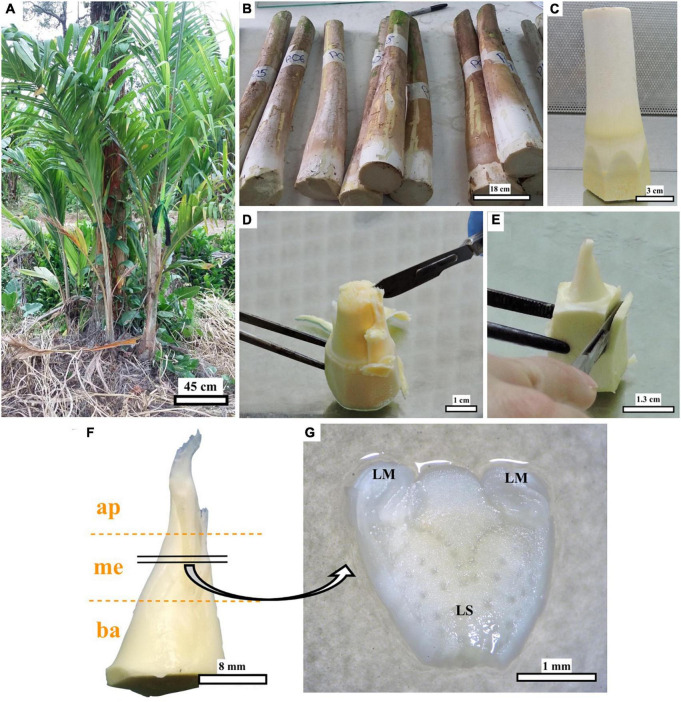
Steps for peach palm explant preparation. Field material **(A)**, offshoots trimmed in the field **(B)**, explant pre-dissected for disinfection **(C)**, further dissection **(D,E)**, position of the explant sections [apical (ap)–medial (me)—basal (ba)] **(F)**, and transverse thin cell layer (explant dissected from each section) on the culture medium with leaf sheath (LS), and leaf sheath margins (LM) marked **(G)**. Black bars scale equivalent to 45 cm **(A)**, 18 cm **(B)**, 3 cm **(C)**, 1 cm **(D)**, 1.3 cm **(E)**, 8 mm **(F),** and 1 mm **(G)**.

Thirty-three offshoots, approximately 1.3 m tall (Figures 1A,B), from adult peach palm (*Bactris gasipaes* Kunth cv. Diamantes 10) plants, growing at the germplasm bank of the Instituto Nacional de Innovación y Transferencia en Tecnología Agropecuaria (INTA), located at the Experimental Station “Los Diamantes” of the Ministry of Agriculture and Husbandry of Costa Rica, located in Guápiles, Limón (latitude: 10.2594, longitude: 83.7715), were used as experimental material. The material was collected in May 2016. Offshoots were trimmed directly in the field to approximately 50 cm in length (Figure 1B) and subsequently cut down in the laboratory to ca. 15 cm in length. These samples had 2–3 layers of leaf sheath (LS) covering the meristematic region (Figure 1C).

### Disinfection and dissection

Explants were scrubbed in the laboratory with cotton balls moistened with ethanol (70% v/v). They were then disinfected individually in glass containers, first by stirring in ethanol (70% v/v) for 2 min and then in a solution of sodium hypochlorite (2% w/v active ingredient) supplemented with two drops of the Tween 80 surfactant (Sigma-Aldrich, St. Louis, MO, USA) per 100 ml, for 35 min. The explants were then rinsed twice with sterile distilled water inside the laminar flow chamber. For dissection, the outer layers covering the meristem were removed with a scalpel and forceps until the explant was approximately 3 cm in length (Figures 1D,E). Subsequently, each explant was divided into three sections of approximately equal size (apical–medial–basal) (Figure 1F). Thin transverse layers about 0.3 mm thick (TCLs) were dissected from each section for culture (Figure 1G) and histological analysis. Transverse TCLs were preferred over longitudinal TCLs because the tissue composition in the former is usually more diverse. This would allow us to increase the responsiveness rate while permitting identifying the particular tissue/explant section responding to the treatments.

### Culture and evaluation

Transverse TCLs were placed with the basal cut in contact with the culture medium composed of the Murashige and Skoog (1962) salts, supplemented with Morel and Wetmore (1951) vitamins, sucrose (30 g L^–1^), glutamine (0.5 g L^–1^), Phytagel*™* (2 g l^–1^) (Sigma-Aldrich, St. Louis, MO, USA), activated charcoal (1.5 g L^–1^) (Steinmacher et al., 2007c; Heringer et al., 2014; Ree et al., 2019), and contained in 9-cm Petri dishes (ca. 25 ml of medium in each). In this phase, fifteen “induction” treatments were tested considering the combination of the three positions of the explant sections and five picloram (4-amino-3,5,6-trichloropicolonic acid) concentrations (150, 300, 450, 600, and 750 μM).

Cultures were placed in dark conditions at 23^°^C–24^°^C. Non-contaminated explants were evaluated and subcultured every 4 weeks on the same medium. Monthly evaluations included contamination, browning, callus, and embryogenic structure formation rates. Subsequently, tissue samples collected from explants showing embryogenic development were examined histologically.

Explants that developed somatic embryos were transferred to the “Development” medium, composed of the basal medium but with a lower picloram concentration (10 μM), contained in 9-cm Petri dishes and under dark conditions at 23–24^°^C. After 2 months, cultures with mature somatic embryos were transferred to the “Conversion” culture medium, in which picloram was replaced by abscisic acid (ABA) (5 μM), also contained in 9-cm Petri dishes and under the same conditions described above. Finally, after two additional weeks, these explants were transferred to the culture medium without plant growth regulators in glass flasks (5.5 cm in diameter and 7 cm in height) and under light conditions (12 h photoperiod, 19 μmol m^–2^ s^–1^) and a temperature of 23–24^°^C, to form plantlets (Steinmacher et al., 2007c; Heringer et al., 2014; Ree et al., 2019).

### *Ex vitro* acclimatization

Plantlets (at least 3 cm tall, from stem base to tip, with more than four roots and at least three developed leaves) were selected for acclimatization. All traces of the culture medium were carefully removed from the roots. The plants were subsequently placed in plastic pots containing peat moss (Jeffy Products International B.V., Moerdijk, Netherlands) and perlite (Specialty Vermiculite Corp., Florida, USA) in a 2:1 ratio. The acclimatization process was carried out in a chamber covered with plastic film (25°C and 74.6% relative humidity). Watering was done manually, once a week, avoiding excess moisture in the substrate. Two weeks later, the plants were fertilized with a foliar spray of Multimineral (2.5 ml L^–1^) (Albion Laboratories Inc., Utah, USA) and N-Control (10 ml L^–1^) (Drexel Chemical Company, Memphis, USA). At that same moment, 0.3 g L^–1^ Benomyl (50 WP, Cafesa, Costa Rica) was sprayed on the acclimatized plants.

### Histological analyses

Initial explants and embryo clusters were sampled several times for histological examination. Tissues were first immersed in a formalin–acetic acid–alcohol (FAA) solution for 8 days at room temperature. FAA was removed by rinsing the tissues with distilled water for 5 min at 67^°^C. Tissue dehydration was conducted through a gradient of ethanol dilutions (60, 70, 80, and 90% v/v at 67^°^C for 5 min each). Further dehydration was then performed by immersing the samples in: ethanol (95% v/v) at 67^°^C for 10 min; ethanol (100% v/v) at 67^°^C for 10 min (repeated twice); a 1:1 solution of ethanol/xylol at 67^°^C for 10 min; two back-to-back solutions of xylol at 67^°^C for 10 min; a 1:1 solution of xylol/paraffin at 60^°^C for 60 min; and finally, two consecutive immersions in a paraffin/paraplast solution at 60^°^C for 60 min. Tissues (frozen blocks) were subsequently sliced 5 μm thick with a rotary microtome (American Optical 820, Buffalo, USA). Paraffin was removed from the tissues using xylol and ethanol dilutions. Tissues were stained with toluidine blue (1% w/v) for 20 min, followed by 15 s wash with tap water. Finally, the samples were dehydrated and rinsed by immersing them for 2 min in ethanol (95% v/v), ethanol (100% v/v), ethanol (100% v/v), and xylol solutions. Finally, under xylol immersion, resin (Paraplast, Permount) was added to each sample (one at a time) and a coverslip was placed over each one (Benavides-Acevedo, 2021). Slides were examined with an inverted microscope Vert A1 (Zeiss, Goettingen, Germany) and with a Leica DM500 light microscope (Leica Microsystems, Heerbrugg, Switzerland). Images of the most representative preparations were captured with a Leica ICC50 digital camera (Leica Microsystems, Heerbrugg, Switzerland). GIMP-2.10 was used to adjust contrast and brightness.

### Statistical analysis

A one-way chi-square test was used to determine whether the frequencies in each treatment (explant position or picloram concentration) were statistically different from each other at each evaluation date. A chi-square test was conducted for results involving the development of primary callus and somatic embryos, as well as of explant browning. Subsequently, for the comparison of means, data were analyzed using Tukey’s test with 95% reliability. All analyses were performed with R software, version 4.0.3 (R Core Team, 2020).

At least nine replicates were used in each treatment (explant position or picloram concentration), with three explants per replicate. Fifteen “induction” treatments were tested considering the combination of the three-explant positions (apical, medial, and basal) and five picloram concentrations (150, 300, 450, 600, and 750 μM).

## Results

### Explant histology and callus induction and characterization

**FIGURE 2:**
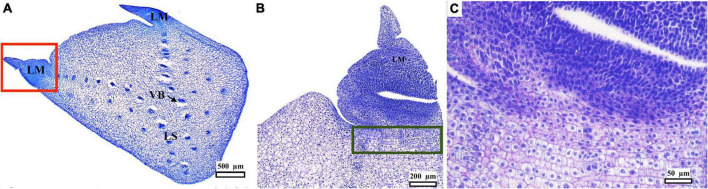
Histological analysis of a peach palm transverse section dissected from initial explants (Figure 1F). Medial transverse section, with leaf sheath margin (LM), leaf sheath (LS), in concordance with Figure 1G, and vascular bundles (VB) **(A)**. Magnification of LM (red square) showing dense cells in the LM and denoting the responsive and competent tissue beneath its base (green rectangle) **(B)**. Further magnification of the responsive zone just underneath the LM **(C)**. Black bars scale equivalent to 500 μm **(A)**, 200 μm **(B),** and 50 μm **(C)**.

Explants (dissected offshoot tips, Figure 1F) were histologically characterized before culturing the TCLs. Transverse sections show details of the general structure of the cultured elements (Figure 1G), with the leaf sheath margins (LMs) located on both sides of the LS (Figure 2A). The distribution of the vascular bundles (VB) in the LS showed a pattern oriented toward the LM (Figure 2A). The LM showed enhanced density of smaller cells (darker tone in Figures 2B,C). It is important to emphasize that it was in the area marked with a green box in Figure 2B that embryogenic structures arose in response to the different treatments, as will be detailed later.

**FIGURE 3:**
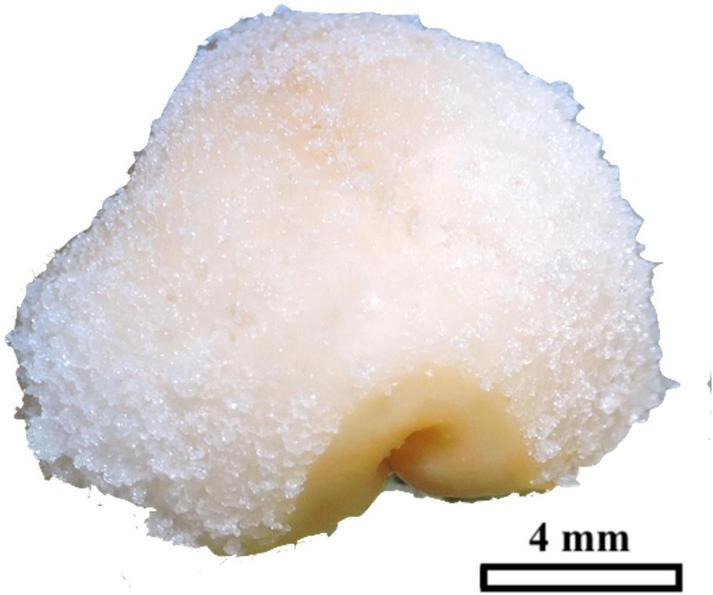
Peach palm primary callus formed on swollen TCLs, consisting of white and fibrous tissues. The black bar scale equivalent to 4 mm.

**FIGURE 4:**
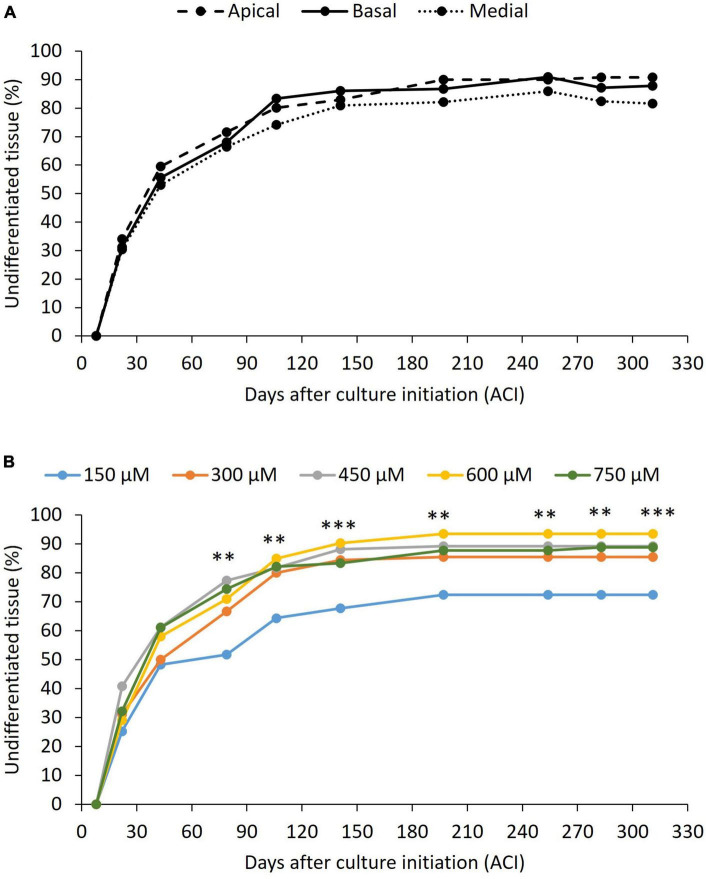
Effect of the peach palm TCLs position **(A)** and picloram concentration **(B)** on undifferentiated tissue formation. *p*-value less than 0.01 (^**^) and less than 0.001 (^***^), according to Tukey’s test at 95% confidence level. Exact *p*-values and differences among treatments can be found in Supplementary material. At least nine replicates per treatment were analyzed.

All explants, regardless of treatment (position or picloram concentration), started to increase in volume on day 22 after culture initiation (ACI). At the same time, some of them also began to form undifferentiated tissue (primary callus). This primary callus was white, fibrous, contained disorganized globular structures (Figure 3), and developed on the surface of the LS of swollen explants. Callus formation increased continuously over time until a steady phase (with 80–90% of explants having developed callus) was reached 197 days ACI (Figures 4A,B). There was no significant relationship between the original position of the explant in the growth axis (apical, medial, or basal) and the rate of callus formation (Figure 4A). The effect of picloram concentration was only evident at 79 days ACI when the explants on the treatment with 150 μM picloram began to show lower callus formation rates than the other treatments. This trend was maintained until the end of the experiment (Figure 4B). The fungal contamination of the explants was mainly detected during the first 8 days ACI and did not exceed 15% (63 out of 405). Additional contamination was sporadically detected from day 79 to 311 ACI (a further 37 explants).

**FIGURE 5:**
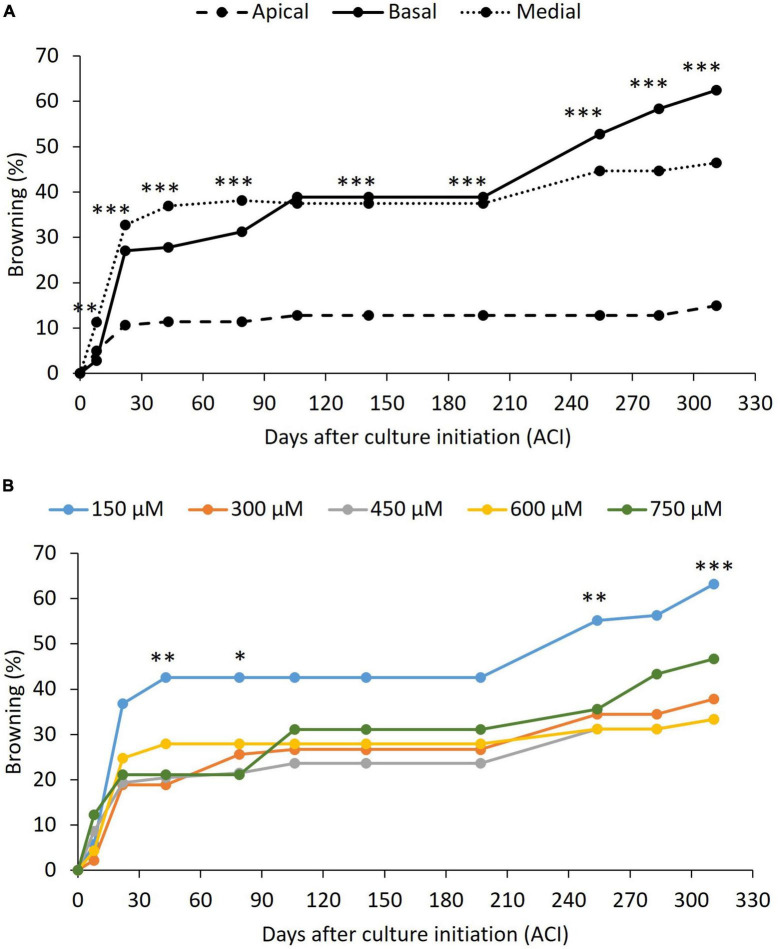
Effect of the peach palm TCLs position **(A)** and picloram concentration **(B)** on browning of explants. *p*-value less than 0.05 (*), less than 0.01 (^**^), and less than 0.001 (^***^), according to Tukey’s test at 95% confidence level. Exact *p*-values and differences among treatments can be found in Supplementary material. At least nine replicates per treatment were analyzed.

Browning only partially affected the explants and was not lethal in any case. It began to be evident 8 days ACI in all treatments. From day 22, an effect of both the explant position and the picloram concentration on the rate of explants showing browning was observed (Figures 5A,B). The absence of significant interactions between the two factors (*p* > 0.05), meaning that picloram concentration had the same effect independently of explant position, compelled us to perform the *post-hoc* tests for each factor independently. Apical explants consistently showed lower browning rates than medial and basal ones throughout the 311-day evaluation period (Figure 5A). In contrast, the lowest picloram concentration showed the highest browning rate of all treatments (Figure 5B). In some explants that showed browning, callus and/or embryo formation occurred as well, meaning that they should not be discarded upon the appearance of these symptoms.

### Induction, development, and characterization of somatic embryos

**FIGURE 6:**
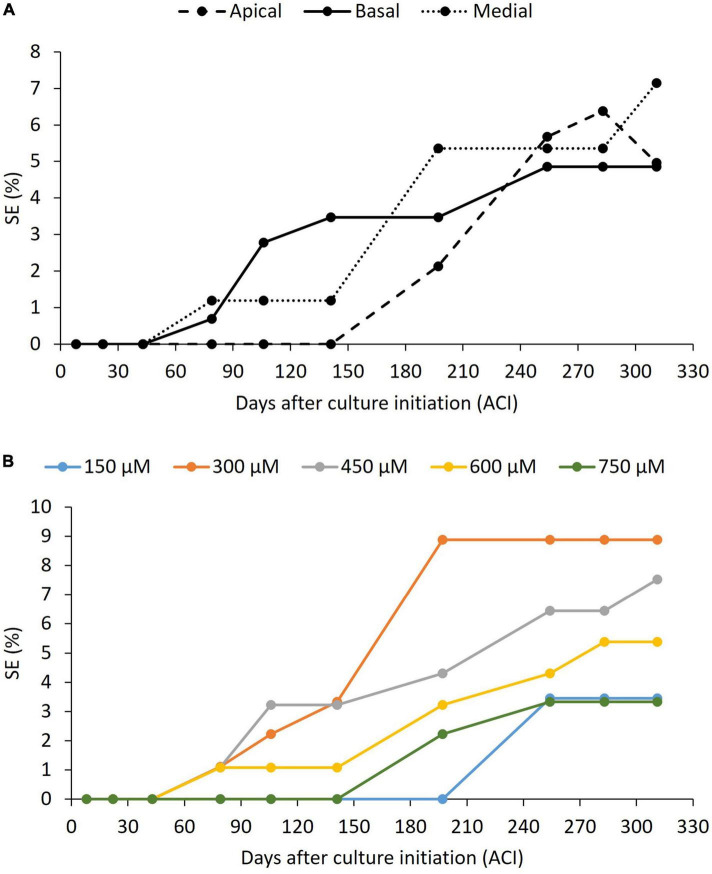
Effect of the peach palm TCLs position **(A)** and picloram concentration **(B)** on induction of somatic embryogenesis (SE). *p*-value was above 0.05 in all cases according to chi-square test at 95% confidence level. Exact *p*-values can be found in Supplementary material. At least nine replicates per treatment were analyzed.

The formation of embryogenic structures was observed on explants derived from the three positions on the growth axis (basal, medial, and apical) (Figure 6A) and with all the evaluated concentrations of picloram (Figure 6B), without any statistically significant difference between them. Of the total explants introduced, 5% (3–9% depending on the treatment) formed embryos within the time period evaluated (311 days ACI). A delay in the onset of the embryogenic response was evident for explants from the apical region (later than 141 days ACI) compared to those from the other two regions (79 days ACI), and of explants at the 150 and 750 μM concentrations of picloram, compared to the other concentrations (Figures 6A,B).

**FIGURE 7:**
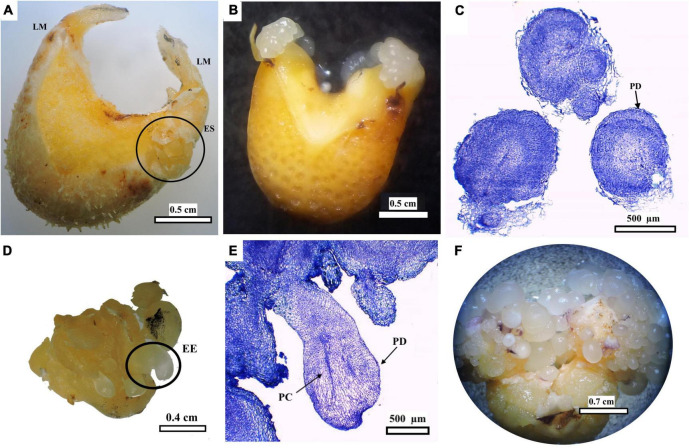
Induction and development of peach palm embryogenic cultures. Swollen TCL showing location of leaf sheath margins (LM) and of initial embryogenic structures (ES) **(A)**. ES further increased their size causing detachment of LM **(B)**. Histological analysis of globular embryos with protoderm (PD) **(C)**. Elongated embryos (EE) **(D)** Histological sections of EE showing procambium (PC) and PD **(E)** Multiplication of somatic embryos forming embryogenic clusters **(F)**. Black bars scale equivalent to 0.5 cm **(A,B)**, 500 μm **(C)**, 0.4 cm **(D)**, 500 μm **(E),** and 0.7 cm **(F)**.

Embryogenic structures, semi-translucent and compact, formed directly on the swollen TCLs, specifically on the lateral parts of the LS (Figure 7A), where the LM is attached. Further growth of the embryogenic structures led to detachment of LMs if they had not detached before (Figure 7B), 3 months ACI (Figure 6B). This region is marked with a green box in Figure 2B. TCLs that neither formed primary callus nor developed embryogenic structures eventually died. Globular embryos were clearly defined by the protoderm and the absence of vascular connections to the surrounding tissues (Figure 7C). Subsequent development of individual globular embryos produced elongated structures (EE), probably scutellar or coleoptilar embryos (Figure 7D). These embryos also exhibited the presence of protoderm separating embryos from surrounding tissues and the development of procambium (Figure 7E). As explants continued to be cultivated with high doses of picloram (150–750 μM), new embryogenic structures continued arising, probably by secondary somatic embryogenesis, without any further development (without bud or root formation, Figure 7F), 5 months ACI.

### Somatic embryo maturation and plantlet development

**FIGURE 8:**
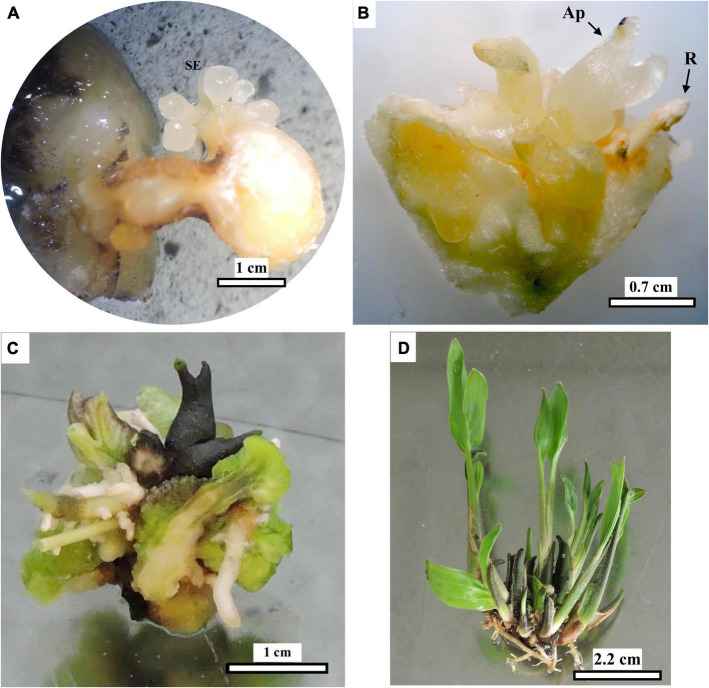
Development and conversion of peach palm somatic embryos into plantlets. Elongated and mature embryos after subculture in the “Development” medium **(A)**, which later developed shoot and root apices when subcultured to “Conversion” medium **(B)**. Explant with shoot and root clusters after transfer to medium devoid of plant growth regulators and to light conditions **(C)**. Rooted plantlet clump after division of larger clump **(D)**. Ap: apical shoot, R root. Black bars scale equivalent to 1 cm **(A)**, 0.7 cm **(B)**, 1 cm **(C),** and 2.2 cm **(D)**.

Subculture of 20 somatic embryo-bearing cultures (Figure 7F) to the “Development” medium resulted in further development of the elongated somatic embryos and globular embryos still present (Figure 8A). Subsequent transfer to the “Conversion” medium resulted in the formation and growth of shoot and root apices (Figure 8B). In addition, transfer to the culture medium devoid of plant growth regulators and light conditions caused the development of additional lateral shoots and roots in 12 of the 20 explants (60% conversion) previously subcultured in the conversion medium, leading to the formation of small shoot clusters (Figure 8C) after approximately 2 months. These embryos followed similar developmental patterns regardless of the original explant’s position and the initial picloram treatment. After dividing some relatively large clumps (with up to 12 shoots), a total of 25 plantlet clumps were obtained (Figure 8D). These clumps had four to 10 roots each. The estimated time to obtain shoot clumps was approximately 13 months ACI, and to obtain complete plantlets ready for acclimatization took approximately four additional months (17 months ACI).

**FIGURE 9:**
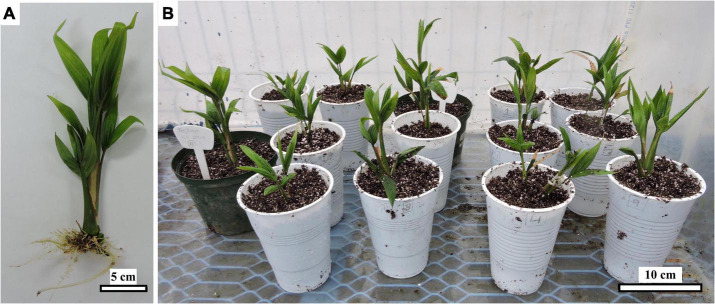
*Ex vitro* peach palm plantlet as comes out of the *in vitro* phase 17 months ACI with 450 μM picloram **(A)** and during acclimatization **(B)**. Black bars scale equivalent to 5 cm **(A)** and 10 cm **(B)**.

Fourteen seedlings were acclimatized under the controlled conditions described in the section “Materials and methods” (Figure 9). However, 3 months after acclimatization, fungal symptoms, probably a consequence of high air relative humidity, were observed at the base of the stems, resulting in 43% mortality.

## Discussion

### Explant anatomy and callus induction and characterization

Peach palm offshoot tips consist of the sheaths or basal sections of the leaves and leaf primordia (unexpanded juvenile leaves and sub-apical tissue), which embrace the stem and will give rise to the future pinnate leaves typical in palms (Steinmacher et al., 2013; Broschat, 2019). Transverse TCLs in this work were dissected from offshoot tips of adult plants (Figure 1), whereas the preceding work of Steinmacher et al. (2007c) used stem sections from *in vitro*-germinated seedlings. Most studies referring to the anatomy of palm meristems show longitudinal sections, where the meristematic dome and leaf primordia can be clearly observed (e.g., Fernando et al., 2003; Jouannic et al., 2011). In our case and in order to better identify from which part of the TCLs the embryogenic structures develop, transverse sections were obtained (Figure 2). A similar pattern for the distribution of the VB toward the LM was also previously described in peach palm (Fonseca et al., 2019). Higher cell density characterizes the LM, which in many cases is separated from the rest of the explant. It is beneath this section (green box in Figure 2B) where the responsive tissue is located, from which the embryogenic structures later develop. The constant division of meristematic cells will eventually promote the formation of primary vascular tissues and organs (Cutler et al., 2008; Jouannic et al., 2011). de Almeida et al. (2012) identified that pre-procambial cells (PPCs), derived from periclinal divisions after 8 weeks in culture, are essential for subsequent competent cell establishment events during peach palm organogenesis and somatic embryogenesis. In our observations, such PPCs are yet to be developed as the histological preparations were done with freshly dissected tissues before tissue culture, aiming at characterizing the original explant.

In contrast to Steinmacher et al. (2007c), who observed a strong effect of the picloram levels between 150 and 600 μM and of the explant position on peach palm primary callus induction, in the present work this effect was almost negligible. Only 150 μM picloram showed a significant reduction in primary callus induction (Figure 4B). Furthermore, in the present work, primary callus induction was observed 1–2 weeks earlier than in the report of Steinmacher et al. (2007c) (22 vs. 28–35 days ACI, respectively). These differences might be associated with a possible effect of the phenological stage of the explants [juvenile in Steinmacher et al. (2007c) vs. mature in our case]. Considering that juvenile explants are often preferred over more developed ones for tissue culture experiments, as young tissues usually respond more readily and more quickly to *in vitro* culture than those coming from aged plants (Weckx et al., 2019), the genetic background of the materials should not be overlooked in this case. The existence of genotypes more responsive and others less responsive to certain culture conditions is known to occur both in monocotyledonous and dicotyledonous species (reviewed by Jiménez, 2005) and was also described in somatic embryogenesis of peach palm (Steinmacher et al., 2007b), even with close-related genotypes (Steinmacher et al., 2013), as in our case [Diamantes-10 derives from Yurimaguas (Arroyo and Mora-Urpí, 2002), the plant material used by Steinmacher et al. (2007c) for their experiment].

The relatively low levels of contamination observed during the induction phase could be related to the degree of protection that the shoot tip receives from the various layers of leaf sheaths, although the presence of endophytes, as described by de Almeida et al. (2005), cannot be out-ruled. The subsequent appearance of contaminants later (progressively after 79 days ACI) could also be related to manipulation during subcultures.

Dissection of TCLs, which are explants with a very high surface-to-volume ratio, causes mechanical cell damage to a high proportion of the explant cells (Teixeira-Da Silva and Dobránszki, 2019). Wounding also brings compartmentalized cell contents into contact with each other, increasing oxidative damage by enzymatic reactions and rising levels of reactive oxygen species (Cassells and Curry, 2001). This could be reflected in the relatively high levels of browning observed in the present work (Figure 5), which exceeded those reported for transverse slices of peach palm inflorescences (<33%, Steinmacher et al., 2007b) and cryopreserved zygotic embryos (<46%, Steinmacher et al., 2007d). The lower browning observed in apical TCLs of peach palm agrees with the results of Al-Khayri (2018), who found less darkening in younger date palm tissues, which was related by Abohatem et al. (2011) to lower phenol content and decreased peroxidase activity in the same species.

Explants that produced less callus (150 μM picloram, Figure 4B) also darkened more frequency (Figure 5B), which is consistent with published evidence (reviewed by Viñas and Jiménez, 2011). However, it was also observed that darkened explants of peach palm could form a callus and even embryogenic structures, as has been reported for date palm (Al-Khayri, 2018), and has even been proposed to be a requirement for callus development in African oil palm (Almeida et al., 2020). Therefore, darkened or oxidized peach palm explants should not be discarded *a priori*, as they may still be responsive.

### Induction, development, and characterization of somatic embryos

The absence of a significant effect of the picloram concentration and the original position of the explant in the growing axis on the induction of embryogenic structures (Figure 6) contrasts with the results of Scherwinski-Pereira et al. (2010) in oil palm and of Steinmacher et al. (2007c) in the peach palm. This may be related, as mentioned above, to the fact that we used adult explants, while the other two referred works used juvenile explants. In addition, Steinmacher et al. (2007c) obtained their explants from *in vitro*-germinated seedlings, a particular growing environment that could also have played a role. The earlier induction of the first somatic embryos (at 79 days ACI in our work) vs. the 140 days ACI required in the work of Steinmacher et al. (2007c) also shows an improved response that should not be overlooked because embryogenic development in some palm species has been categorized as a lengthy process (Pfenning et al., 1998). However, the best embryogenic response in our work was only one-fifth of the best one obtained by Steinmacher et al. (2007c) with 300 μM picloram and medial peach palm TCL explants, and one-fourth of that of Scherwinski-Pereira et al. (2010) with 450 μM picloram and basal African oil palm TCL explants. This may also be attributed to differences related to explant juvenility and genotype as detailed above.

Steinmacher et al. (2007c) also observed clearly discernible globular somatic embryos developing directly beneath the LM boundary. The location of this responsive zone may be favored by the constant division of the meristematic cells present there, which eventually promotes the formation of primary vascular tissues, leading subsequently to the formation of embryogenic structures (Cutler et al., 2008; Jouannic et al., 2011). The protoderm, separating the somatic embryos from the surrounding tissues, could also be observed (Figures 7C,E), similar to previous findings in somatic embryogenesis of peach palm (Steinmacher et al., 2007a,c) and other palm species (Moura et al., 2008; Silva et al., 2014). As they developed, some embryos elongated and the presence of procambium was more evident in their central zone, again without obvious vascular connection to the mother explant (Figure 7D). These signs of embryo polarization have been reported previously in peach palm (Steinmacher et al., 2007a,c, 2011; Padilha et al., 2015). Continuous culture with picloram arrested any further progression in the development of the peach palm somatic embryos. Still available embryos began to divide through secondary somatic embryogenesis, similar to what was observed for this same species (Steinmacher et al., 2011) and for *Acrocomia* palm (Moura et al., 2009).

### Somatic embryo maturation and plantlet development

In general, after the maturation and conversion phases, the initial development of shoots and roots took place in the culture medium free of plant growth regulators and under light conditions, until complete plantlets were obtained (Figure 8), as also reported by Steinmacher et al. (2007b,c). Formation of shoot clumps (more than one growing axis per agglomerate) evidenced part of the advantages of using somatic embryogenesis in palms because of the possibility of obtaining more than one plant from each dissected explant (Viñas and Jiménez, 2011; Steinmacher et al., 2013). This is a relevant alternative for *in vitro* propagation of plants that present difficulties for conventional propagation, such as peach palm, to reduce the establishment time of elite plants and to set up conservation and rescue programs for genotypes with desirable characteristics (Steinmacher et al., 2007c; Viñas and Jiménez, 2011; Ree and Guerra, 2015). Plant death during acclimatization is not a rare event. This phase has been considered a bottleneck for plant micropropagation (Hassan et al., 2014), particularly in peach palm (Steinmacher et al., 2007c). Acclimation could be improved by supplementing the substrate with MS nutrients and by immersing the roots in fungicide solutions, as was done in date palm (Hassan et al., 2021). The results presented in this work are very promising, as they demonstrate the feasibility of mass propagation of adult peach palm elite plants from lateral offshoots by somatic embryogenesis from transverse TCL explants. This protocol could also be of great value for rescuing individuals conserved in *ex vitro* germplasm collections to avoid gene erosion due to plant aging and mortality.

## Data availability statement

Chi-square and mean comparisons are included in the article/Supplementary material. Further inquiries can be directed to the corresponding author.

## Author contributions

SC-B and MV: conceptualization, methodology, investigation, formal analysis, and writing—original draft, review and editing. PS-C and AH: methodology, formal analysis, and writing—review editing. DAS: methodology and writing—review and editing. MPG: conceptualization and writing—review and editing. VMJ: conceptualization, formal analysis, writing—original draft, review and editing. All authors contributed to the article and approved the submitted version.
